# Does Chicago Classification address Symptom Correlation with High-resolution Esophageal Manometry?

**DOI:** 10.5005/jp-journals-10018-1231

**Published:** 2017-09-29

**Authors:** Mayank Jain, Melpakkam Srinivas, Piyush Bawane, Jayanthi Venkataraman

**Affiliations:** 1 Department of Gastroenterology, Gleneagles Global Health City, Chennai, Tamil Nadu, India,; 2 Department of Gastroenterology, Choithram Hospital and Research Centre, Indore, Madhya Pradesh, India

**Keywords:** Classification, Dysphagia, Esophagus, Motility, Pain.

## Abstract

**Aim:**

To assess the correlation of symptoms with findings on esophageal high-resolution manometry (HRM) in Indian patients.

**Materials and methods:**

Prospective data collection of all patients undergoing esophageal manometry was done at two centers in India—Indore and Chennai—over a period of 18 months. Symptom profile of the study group was divided into four: Motor dysphagia, noncardiac chest pain (NCCP), gastroesophageal reflux (GER), and esophageal belchers. The symptoms were correlated with manometric findings.

**Results:**

Of the study group (154), 35.71% patients had a normal study, while major and minor peristaltic disorders were noted in 31.16 and 33.76% respectively. In patients with symptoms of dysphagia, achalasia cardia was the commonest cause (45.1%), followed by ineffective esophageal motility (IEM) (22.53%) and normal study (19.71%). In patients with NCCP, normal peristalsis (50%) and ineffective motility (31.25%) formed the major diagnosis. Of the 56 patients with GER symptoms, 26 (46.4%) had normal manometry. An equal number had ineffective motility. Of the 11 esophageal belchers, 7 (63.6%) of these had a normal study and 3 had major motility disorder. Dysphagia was the only symptom to have a high likelihood ratio and positive predictive value to pick up major motility disorder.

**Conclusion:**

Dysphagia correlates with high chance to pick up a major peristaltic abnormality in motor dysphagia. The role of manometry in other symptoms in Indian setting needs to be ascertained by larger studies.

**Clinical significance:**

The present study highlights lack of symptom correlation with manometry findings in Indian patients.

**How to cite this article:** Jain M, Srinivas M, Bawane P, Venkataraman J. Does Chicago Classification address Symptom Correlation with High-resolution Esophageal Manometry? Euroasian J Hepato-Gastroenterol 2017;7(2):122-125.

## INTRODUCTION

The esophageal motor function is evaluated using various techniques, including barium radiography, radionuclide transit studies, manometry with or without impedance testing, and more recently impedance planimetry.^1^ Chicago classification 3.0 is the latest that is used across the world for classifying and management of esophageal motor disorders.^2^ Esophageal motility study using water perfused high-resolution esophageal manometry (HREM) provides information on physiological function of the esophagus and attempts at correlating the esophageal function with the symptoms. Mikaeli et al^3^ have reported a good correlation between the intensity of the motor abnormality and symptom severity in achalasia.^4^ Fakhre Yaseri et al^5^ found HREM to be a useful tool in diagnosis of achalasia. It could differentiate achalasia from those who had normal esophageal motility.

The diagnostic value of water perfusion HREM for indications other than achalasia, e.g., esophageal symptoms like dysphagia, GER disease is still unknown.^5^ In our day-to-day practice, we encounter difficulty in correlating HREM findings with the clinical presentation. We therefore retrospectively correlated the HREM findings with symptoms in patients with dysphagia, NCCP, and GER disease.

## MATERIALS AND METHODS

In this study, 187 patients from two centers—Choithram Hospital and Research Centre, Indore (MJ) and Gleneagles Global Health City, Chennai (MS, PB, VJ)— who had HREM between July 2014 and December 2015 were analyzed. Patients with one or more combination of symptoms of dysphagia, NCCP, regurgitation, and heartburn were included.

Demographic data including age, gender, and symptoms of the patients were noted in detail. High-resolution manometry was done and reported by gastroenterologists with more than 2 years of experience with HRM. Manometry was done in supine position using 16-channel water perfusion systems (Ready Stock, Australia). The studies were conducted with the patient in the supine position after at least a 6-hour fast, and medications that could affect the esophageal motor function (e.g., metoclopramide, anticholinergics, and smooth muscle relaxants) were discontinued for 5 to 7 days prior to the study. After application of topical anesthetic into the nostril, the manometry catheter was passed transnasally and the sensors were positioned to record from the hypo pharynx to the stomach. After the lower esophageal sphincter (LES) was detected via the stationary pull-through method, the catheter was fixed in place by taping it to the nose. Basal LES pressures were recorded for 1 minute. Wet swallows were done using ten swallows of 5 mL each. The data were reported as per Chicago Classification v 3.0. Patients below 18 years of age, previous foregut surgery, large hiatus hernia, esophagitis of grade C or D, eosinophilic or candidal esophagitis, Barrett’s esophagus, and cardiovascular disease were excluded.

The correlation of following symptoms was assessed with the final manometry diagnosis: Dysphagia for both solids and liquids with or without weight loss, suggestive of motor dysphagia; NCCP; reflux symptoms; regurgitation and/or heartburn with or without NCCP; GER; and esophageal belchers; belchers.

The correlation of above-mentioned symptoms to an identifiable manometric abnormality (as per major and minor peristaltic defects, Chicago v 3.0) was assessed by calculating sensitivity, specificity, positive likelihood ratio, negative likelihood ratio, and positive and negative predictive values.

## RESULTS

A total of 187 patients from two centers underwent HRM during the study period. Based on the fulfilment of inclusion criteria, 154 patients were included in the study. The male:female ratio was 1.32:1 and median age was 45.5 years (18-77 years). Of the study group, 55 (35.71%) patients had a normal study, while major and minor peristaltic disorders were noted in 31.16 and 33.76% respectively. Seventy-one patients presented with motor dysphagia. Achalasia cardia was the commonest cause (45.1%), followed by IEM (22.53%) and normal study (19.71%).

**Graph 1:**
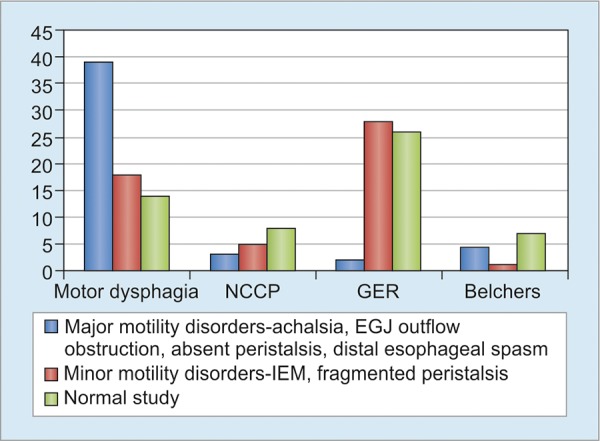
Manometry diagnosis with respect to major symptoms studied

Sixteen patients presented with NCCP. Among these patients, normal peristalsis (50%) and ineffective motility (31.25%) formed the major diagnosis after manometric evaluation. Major motility disorders, like achalasia cardia and absent peristalsis, were noted in three cases (18.75%). Of the 56 patients with GER symptoms, 26 (46.4%) had normal manometry. An equal number had ineffective motility. Fragmented peristalsis and absent contractility were noted in two cases (3.5%) each. None of these patients had dysphagia. Of the 11 esophageal belchers, 7 (63.6%) of these had a normal study. However, 2 patients had achalasia cardia (18.18%) and 1 each (9.1%) had ineffective motility and absent contractility. The manometry diagnosis with respect to the major symptoms studied is shown in Graph 1. The sensitivity, specificity, positive likelihood ratio, negative likelihood ratio, positive and negative predictive values for all the symptoms are shown in Table 1.

## DISCUSSION

Manometry is regarded as gold standard investigation of motility disorders in the esophagus. The transport of bolus is successful when minimal bolus material is retained within the esophageal body.^6^ However, numerous factors that may affect transit and motility, such as bolus shape, surface, and consistency cannot be measured with manometry.^7^ Dysphagia and NCCP are considered to be the predominant symptoms in motility disorders. Regurgitation, heartburn, hoarseness, and asthma are hallmark of reflux disease.^8^ The present study was done to ascertain the correlation of symptoms and findings noted in esophageal manometry in Indian setting. While most patients presenting with dysphagia had a demonstrable abnormality of manometry, most patients with NCCP had a normal study. In patients with reflux symptoms, minor peristaltic abnormalities and normal manometry were the common reported findings. Interestingly, in the subset of esophageal belchers, we picked up major motility disorders in one-third of cases. In our group of patients, dysphagia for solids and liquids was the only symptom to have a higher likelihood of showing peristaltic abnormality. Symptoms of dysphagia had a high positive predictive value for detecting dysmotility. None of the other symptoms assessed showed a good positive predictive value or likelihood for detection of peristaltic abnormality. There are a few reports of symptom correlation with esophageal manometry outcomes. Saha et al^9^ reported that manometry showed abnormalities in 54.83, 68.42, and 38.70% of patients with reflux, dysphagia, and NCCP respectively. In reflux group, the most common abnormalities were hypoperistalsis (22%), followed by hypotensive LES (29.67%). In patients with dysphagia, the most common motor abnormality was achalasia cardia (55.26%), whereas in case of chest pain, hypotensive LES was frequently noted.^10^ Our study findings are similar to reported in other studies where it was noted that manometry yields a higher diagnostic value in patients with dysphagia. Manometry is not a first-choice functional diagnostic test in the study of patients with GER or NCCP.^10^ Gambitta et al,^11^ however, highlighted the role of manometry in symptoms of dysphagia, chest pain, and refractory reflux disease. Ineffective esophageal motil-ity is an important cause of symptoms in patients with chest pain, dysphagia, and reflux symptoms. It is also a minor esophageal motility disorder characterized by weak esophageal contractions that may result in abnormal esophageal bolus clearance. The clinical significance and outcome of this subgroup is not clearly known. However, few case reports of progression to major peristaltic abnormalities like esophageal spasm and achalasia have been reported in the literature.^12^ The changes of IEM are persistent even on long-term follow-up.^13^ Other authors have reported that esophageal symptoms may be secondary to esophageal hypersensitivity^14^ or to factors beyond the circular muscle contraction patterns,^15^ which at present cannot be fully estimated using HRM. The present study highlights the role of manometry in our setting in India. While HRM seems to have a definite role in evaluation of dysphagia, its role in evaluation of NCCP and belching remains to be studied. In reflux patients, we did not find any major peristaltic abnormality and this affirms our belief that esophageal manometry may not be required for all reflux patients. However, its role in refractory cases and prior to fundoplication remains to be studied in our setting. Our study has few limitations in view of small sample size and the fact that motility patterns may differ between liquid and solid boluses.

**Table 1:** **Table 1:** Correlation of symptoms with peristaltic abnormality on HRM

*Symptoms*		*Sensitivity (95% CI)*		*Specificity (95% CI)*		*Positive likelihood ratio (95% CI)*		*Negative likelihood ratio (95% CI)*		*Positive predictive value (95% CI)*		*Negative predictive value (95% CI)*	
Motor		57.58%		74.55%		2.26		0.57		80.28%		49.40%	
dysphagia		(47.23-67.45)		(61-85.33)		(1.40-3.67)		(0.43-0.57)		(71.53-86.84)		(42.54-56.28)	
NCCP		8.08%		85.45%		0.56		1.08		50%		34.06%	
		(3.55-15.30)		(73.34-93.50)		(0.22-1.40)		(0.95-1.22)		(28.44-71.56)		(31.34-36.89)	
GER		30.30%		52.73%		0.64		1.32		53.57%		29.59%	
		(21.47-40.35)		(38.8-66.35		(0.43-0.96)		(1-1.75)		(43.44-63.46)		(24.07-35.78)	
Belchers		4.04%		87.27%		0.32		1.10		36.36%		33.57	
		(1.11-10.02)		(75.52-94.73)		(0.10-1.04)		(0.99-1.23)		(14.89-65.11)		(31.19-36.03)	

CI: Confidence interval

## CONCLUSION

Dysphagia correlates with high chance to pick up a major peristaltic abnormality in motor dysphagia. The role of manometry in other symptoms in Indian setting needs to be studied further. The significance of these manometry findings in terms of patient care and pharmacotherapy needs an in-depth evaluation. So the final question remains: Do we treat symptoms or do we treat the physiological changes at HRM which do not have significant correlation with patients symptoms? It is time that Chicago classification dwells and addresses these issues.

## CLINICAL SIGNIFICANCE

Apart from dysphagia, none of the other common symptoms predict the presence of a major peristaltic abnormality in Indian patients.
